# Western Ontario Veterinary Association

**Published:** 1895-03

**Authors:** 


### WESTERN ONTARIO VETERINARY ASSOCIATION.

A very important meeting of the Western Ontario Veterinary Association was held at Listowel, Ontario, January 24 and 25, 1895. President Burger occupied the chair, and, calling for the report of the Committee on Resolutions regarding the curriculum and management of the Ontario Veterinary College, the following was presented:

That a matriculation examination be established, requiring applicants to read selected passage from ordinary English prose, to write legibly and correctly an English composition; to write English prose from dictation, and to understand the simple and compound rules of arithmetic, including decimal fractions.

That the Board of Entrance Examiners be appointed by the Agriculture and Arts Association and Veterinary Council respectively, two members each.

That the term of tuition be extended to three years, of five months each, exclusive of examinations and Christmas vacations.

That the Board of the Agriculture and Arts Association appoint the examiners, subject to the approval of the Western Ontario Veterinary Medical Association, and that they be selected from resident veterinary surgeons of Ontario, and that every student must practice during vacations with a qualified practitioner and produce certified certificates to that effect.

Mr. W. Gibb, of St. Mary's, speaking on the subject-matter of the resolutions, said that this school was started in 1862 on a small scale, with few students. That the college is the property of Prof. Andrew Smith, and the profits arising therefrom reach eight to ten thousand dollars a year. The staff composed of Prof. Smith, V.S., J. Thorburn, M.A., Michael Barrett, M.D., T. Babington, and J. T. Duncan, M.D., V.S. That the whole or part of Mr. Duncan's salary as lecturer is paid by the Agriculture and Arts Association of Ontario. The studeuts pay fees to Mr. Babington for his lectures on chemistry. Dr. Smith has to pay himself and Drs. Barrett and Duncan.

The practice of veterinary medicine in the province of Ontario is controlled by the Agriculture and Arts Association, it holding examinations and granting diplomas to practise. He charged that virtually the whole matter was controlled by Prof. Smith; that the annual announcement did not give sufficient particulars of the college; that no matriculation is required, and as consequence the profession suffered throughout the province. He urged that all candidates be required to pass a severe matriculation examination, and quoted the requirements of the veterinary department of Harvard and the University of Pennsylvania. The short course at Toronto, he said, was why so many American students registered there. He urged the extension of the curriculum, and said that the live-stock interests of to-day demanded higher skilled practitioners.

J. Wilson, V.S., of Wingham, after referring to the income of the college and the small proportionate amount used for the employing of instructors, charged that the whole efforts seemed to be to increase the monetary income of the school, and that it should be changed from top to bottom.

After further discussion the report of the Committee on Resolutions was adopted.

W. Gibb, V.S., of St. Mary's, read a paper on "Purpura Hemorrhagica," which he treated with cocaine internally, reporting several cases treated, with only one death.

Subsequent to the meeting Prof. Andrew Smith said in defense of the criticisms made, that the institution was under the auspices of the Agriculture and Arts Association, who grant the diplomas; that originally the Board of Agriculture contributed to the expense of the institution, but at present he bore all the expense personally. He estimated the cost of the present buildings at $60,000, and that the facilities and course of study was unequalled on this continent, and he claimed it to be the best institution in America. That many of its graduates are teachers and professors of veterinary science in agricultural colleges in Canada and the United States. Of the one hundred and forty junior students at present attending, only thirty-seven came from Ontario. He said the curriculum was equal to any on the continent, and that only a few of the colleges on this side of the water were three-term schools, and that the colleges on the other side of the water were in the same position, whether of London, Glasgow, or Edinburgh. That some of those are under the control of boards of trustees, but others are conducted on the same basis as the Ontario Veterinary College.

